# BlackOPs: increasing confidence in variant detection through mappability filtering

**DOI:** 10.1093/nar/gkt692

**Published:** 2013-08-08

**Authors:** Christopher R. Cabanski, Matthew D. Wilkerson, Matthew Soloway, Joel S. Parker, Jinze Liu, Jan F. Prins, J. S. Marron, Charles M. Perou, D. Neil Hayes

**Affiliations:** ^1^Department of Statistics and Operations Research, University of North Carolina, Chapel Hill, NC 27599, USA, ^2^The Genome Institute at Washington University, St. Louis, MO 63108, USA, ^3^Lineberger Comprehensive Cancer Center, University of North Carolina, Chapel Hill, NC 27599, USA, ^4^Department of Genetics, University of North Carolina, Chapel Hill, NC 27599, USA, ^5^Department of Computer Science, University of Kentucky, Lexington, KY 40506, USA, ^6^Department of Computer Science, University of North Carolina, Chapel Hill, NC 27599, USA and ^7^Division of Medical Oncology, Department of Internal Medicine, University of North Carolina, Chapel Hill, NC 27599, USA

## Abstract

Identifying variants using high-throughput sequencing data is currently a challenge because true biological variants can be indistinguishable from technical artifacts. One source of technical artifact results from incorrectly aligning experimentally observed sequences to their true genomic origin (‘mismapping’) and inferring differences in mismapped sequences to be true variants. We developed BlackOPs, an open-source tool that simulates experimental RNA-seq and DNA whole exome sequences derived from the reference genome, aligns these sequences by custom parameters, detects variants and outputs a blacklist of positions and alleles caused by mismapping. Blacklists contain thousands of artifact variants that are indistinguishable from true variants and, for a given sample, are expected to be almost completely false positives. We show that these blacklist positions are specific to the alignment algorithm and read length used, and BlackOPs allows users to generate a blacklist specific to their experimental setup. We queried the dbSNP and COSMIC variant databases and found numerous variants indistinguishable from mapping errors. We demonstrate how filtering against blacklist positions reduces the number of potential false variants using an RNA-seq glioblastoma cell line data set. In summary, accounting for mapping-caused variants tuned to experimental setups reduces false positives and, therefore, improves genome characterization by high-throughput sequencing.

## INTRODUCTION

A prerequisite to identifying variants from high-throughput sequencing data is to align or ‘map’ a read back to its originating location in the genome. This is a difficult task because of short sequence length, genomic similarity due to homology, sequencing errors and, in the case of RNA-seq, splice junctions (1). While the performance of alignment algorithms continues to improve both in speed and accuracy, there is no aligner that has perfect sensitivity and perfect specificity (2,3). That is, none of the current alignment algorithms can exactly map each experimental sequence to its true location in the genome. At current short read lengths (50–100 nucleotides), this lack of perfect alignment will continue to persist. It is important to fully understand why reads are being incorrectly mapped and how mapping errors impact downstream analyses such as variant detection.

The detection and characterization of genomic sequence variation can lead to a better understanding of disease pathology (4) and in some cases to new therapeutic targets (5). Genomic sequence variants are divided into two types based on their origin: germline variants that are inherited, such as single nucleotide polymorphisms (SNPs), and somatic mutations that develop within a person’s cells over time. Recently, there has been an abundance of germline and somatic variant profiling studies that take advantage of new high-throughput sequencing technologies (6,7). Currently, the most popular sequence variant profiling technology is DNA whole exome sequencing (DNA-WES). DNA-WES consists of capturing a predefined set of genome targets corresponding to exons, and then sequencing the resulting captured sequences. DNA-WES has been shown to have high sensitivity and specificity when detecting variants (8,9). Alternatives to DNA-WES include DNA whole genome sequencing (WGS), which is more expensive, and RNA-seq, which is limited to expressed genes. Despite the expression constraint, RNA-seq can detect 70–80% of the exonic variants in well-expressed genes (6,10). Unfortunately, mapping errors can lead to large numbers of false variant calls on these platforms (11–13). Sequence mapping errors can present themselves as ‘unmapped reads’, reads that map to multiple locations (‘multimapped reads’) or reads that map uniquely to only one genomic location but it is an incorrect location (which we will term ‘uniquely mismapped reads’). It is important to differentiate these mapping errors and how they impact downstream analyses because failing to account for such errors can significantly alter the results and interpretation of an entire study (3,14–16). The fact that a single mutation could be relevant to a patient’s treatment strategy makes it imperative that researchers have the computational ability to accurately predict variants while minimizing false variant calls.

Labs that regularly process high-throughput sequencing data are aware of mapping errors and their effect on downstream analyses, yet there is no consensus on how to best account for these errors. Previous studies have explored different aspects of read ‘mappability’, or the likelihood that a read can be mapped to its proper location, and its effect on variant calling (17–19). These mappability tracks cannot easily be extended to RNA-seq because they do not consider reads that span splice junctions, which is a major source of RNA-seq mapping errors (11). Although some groups align RNA-seq data to the genome after ‘masking’ known SNP positions, this strategy has been shown to be ineffective at improving variant calling (14). Mapping errors have also been incorporated into pipelines that identify RNA-editing sites (3,16), but these are transcriptional events and not genomic sequence variants. To date, there is no standardized method or reference for identifying variants that may be caused by mapping errors, leaving each lab to design their own one-off solution.

Here, we sought to investigate the effects that mapping errors might have on variant detection under conditions of different sequence read length, alignment algorithm and profiling assay (DNA-WES and RNA-seq). To achieve this, we developed BlackOPs (‘Blacklist Of Positions’), a publicly available tool that uses simulated reads and outputs a list of sequence variants caused by mapping errors that are indistinguishable from true biological variants. This ‘blacklist’ can be used to filter variant predictions and reduce false positives caused by mapping artifacts. We used BlackOPs to assess the performance of different read lengths and alignment algorithms, characterize reads that are often mismapped and investigate how these mapping errors, when not accounted for, result in a large number of false variant calls, which are sometimes present in variant databases such as dbSNP and Catalogue Of Somatic Mutations In Cancer (COSMIC). Additionally, we demonstrate the utility of our tool by showing that filtering variant calls against the appropriate blacklist can reduce the number of false positives using an RNA-seq glioblastoma cell line data set.

## MATERIALS AND METHODS

### Software and blacklist availability

BlackOPs is open-source software, written in Perl, distributed under GPL-3.0, and available at http://sourceforge.net/projects/rnaseqvariantbl/. All blacklists produced for this manuscript are also available at this site.

### Reference transcriptome and read alignment

The set of genomic coordinates corresponding to the hg19 UCSC known gene transcripts (20) was downloaded from the UCSC genome browser on 16 June 2012. After removing transcripts outside chr 1-22, X and Y, 76 969 transcripts remained. To ensure that the poly-A tail was not included and that each transcript exactly matches a subset of hg19, the sequence for each reference transcript was obtained by extracting the corresponding sequence from the hg19 reference genome sequence. If a transcript was aligned on the negative strand, the reverse complement was used to retain the strand-specific information.

To generate a set of single-end (SE) reads of length *L* [50, 75, 100, 200 base pairs (bp)], each position along a transcript was considered as the start position of a new read and the transcript sequence of length *L* starting at that position was recorded. To guarantee uniform read length, all reads with length less than *L* were removed. Except at the ends of a transcript, each position along the transcript was covered by *L* SE reads. This process was repeated across all reference transcripts. In a similar fashion, a set of paired-end (PE) reads with a fixed insert size of 200 bp was generated. Read identifiers and positions were retained, allowing us to determine the correct genomic location of each read. Note that these simulations are deterministic; there is no randomness associated with generating these reads. Although uniform coverage will not be observed in practice, this simulation allows us to systematically identify all possible mapping errors, which would not be possible if reads were randomly simulated to reflect coverage patterns observed in RNA-seq data.

The simulated reads were aligned to hg19 using the default settings of MapSplice version 2.1.3 (21) and TopHat version 2.0.6 (22). The one exception is that the 2 × 36 reads were aligned after changing the option for segment length to 18. The SE reads were also aligned to the set of UCSC transcripts alone (the transcriptome) using TopHat (setting the –T option) with the mappings reported in genomic coordinates. For the PE analysis, reads with unmapped mates or insert sizes >1 Mb were filtered out and designated as unmapped reads. The location of ‘uniquely mapped reads’ (mapping to only one genomic location) was compared with the known hg19 location where the read originated. If a read did not map within 5 bp of its true hg19 location, it was marked as mismapped. We tolerate a 5 bp shift to not penalize reads where the majority of bases map correctly but a few bases at the end of the read are mismapped. This situation may arise when a read contains a splice junction within a few bases of an end. By this definition, a read that is correctly mapped (i.e. not mismapped) may contain mismatches.

The set of Ensembl paralogous human genes (23) with matching UCSC IDs for reference genome GRCh37.p8 were downloaded on 28 December 2012. This list was used to determine whether uniquely mismapped reads mapped to a known paralog.

### Insertion of sequencing errors

To assess the influence of random sequencing errors on mapping results, random base substitutions were made to the set of 75 bp SE reads. Each position along the read was treated independently and a base was modified to one of the three remaining bases (for example, an A was changed to a C, G or T) with probability of 10^−^^4^. To ensure sufficient coverage so that all splice junctions could be correctly identified by the alignment algorithm, the reads containing an error were aligned to the genome along with the set of reads containing only the reference sequence using TopHat.

### Insertion of known SNPs

SE reads overlapping a SNP [all dbSNP positions, version 131 (24)] were identified and one member of each pair was edited such that the alternate allele (from dbSNP) replaced the corresponding reference allele. Reads were restricted to contain at most one SNP; reads containing two or more SNPs were duplicated so that each read contained only one non-reference allele. This ensured that each SNP position was covered by *L* reads containing the alternate allele (with the exception of positions at the ends of a transcript). Reads without an inserted SNP were filtered out. To ensure sufficient coverage so that all splice junctions could be correctly identified by the alignment algorithm, the SNP-inserted reads were aligned to the genome with the set of reads containing only the reference sequence. After alignment, the reference-only reads were filtered from the analysis.

### Whole exome sequencing analysis

SE reads of length *L* were simulated from the set of 194 680 exon targets of Agilent’s SureSelect Human Exon Kit version 2. The same procedures used for the RNA-seq analysis were repeated for the DNA-WES analysis.

### Identifying single nucleotide differences

Single nucleotide differences (SNDs; genomic positions that are covered by at least one non-reference base) of uniquely mapped reads were identified using VarScan (25) with min-coverage = 1, min-reads2 = 1, min-avg-qual = 0 and min-var-freq = 0. The output was filtered for duplicate genomic positions. Mismatches occurring in exons were determined by intersecting all unique SNDs with the positions listed in hg19 UCSC known gene transcripts. The SNDs were intersected with the following variant lists: all dbSNP positions for versions 131, 132 and 135 in hg19 coordinates and COSMIC version 52 (26). VarScan was also used with the following parameters to identify high coverage positions likely to be called variants: min-coverage = 30, min-reads2 = 10, min-avg-qual = 0, min-var-freq = 0.1 and *P* = 1e-20.

### Cell line analysis

Two replicates of RNA from the U87 glioblastoma cell line (27) were sequenced as part of The Cancer Genome Atlas using Illumina’s Genome Analyzer II, representing identical sequence runs but of slightly differing quality. Each run produced 76-bp SE reads, which were aligned to hg19 plus chrM using MapSplice version 1.15.2. The uniquely aligned reads were sorted and indexed, and the pileup file, both with and without the BAQ option (-B), was created using SAMtools (28). Variants were called using VarScan with the following parameters: min-coverage = 30, min-reads2 = 10, min-var-freq = 0.1 and *P* = 1e-20. Variants from chrM and those not called in both replicates were removed. Positions not overlapping with exons in hg19 UCSC known gene transcripts or reported in dbSNP version 135 were filtered. BlackOPs was run in both the reference-only and SNP-inserted modes using 75 bp SE reads aligned with MapSplice version 1.15.2. Variants were filtered against both the reference-only and SNP-inserted blacklists.

### Statistical analysis

All statistical analyses were performed in R. Paired *t*-tests were calculated using all eight SE data sets (combining both TopHat and MapSplice results). Binomial tests were calculated separately for each of the eight SE data sets, resulting in eight tests. A binomial test was used to determine whether the set of novel variants in the cell line data is significantly enriched for blacklist positions under the null hypothesis that (*x*/*n*) = *p*, where *n* is the total number of variants (129), *x* is the number of variants at blacklist positions (92) and *p* is the proportion of exon positions across the genome listed in the blacklist file (37 540/73 418 700). Figures were created using the R packages VennDiagram (29) and ggplot2 (30).

## RESULTS

### Identification and characterization of RNA-seq alignment errors

To gain an understanding of mapping performance in the absence of sequencing errors and biological variants, reads were generated from the reference genome corresponding to the set of UCSC transcripts. The reference transcripts were computationally split into SE and PE reads of multiple fixed lengths (1 × 50, 1 × 75, 1 × 100, 1 × 200, 2 × 36, 2 × 50, 2 × 75, 2 × 100). Each read length was aligned to the genome using both MapSplice and TopHat, resulting in 16 data sets. This study can be viewed as a ‘best-case scenario’ because each read perfectly matches some subset of the reference genome and, thus, should map without any mismatches. That is, when a read is correctly mapped, every base along the read should match the reference sequence at that position.

**Figure 1.**
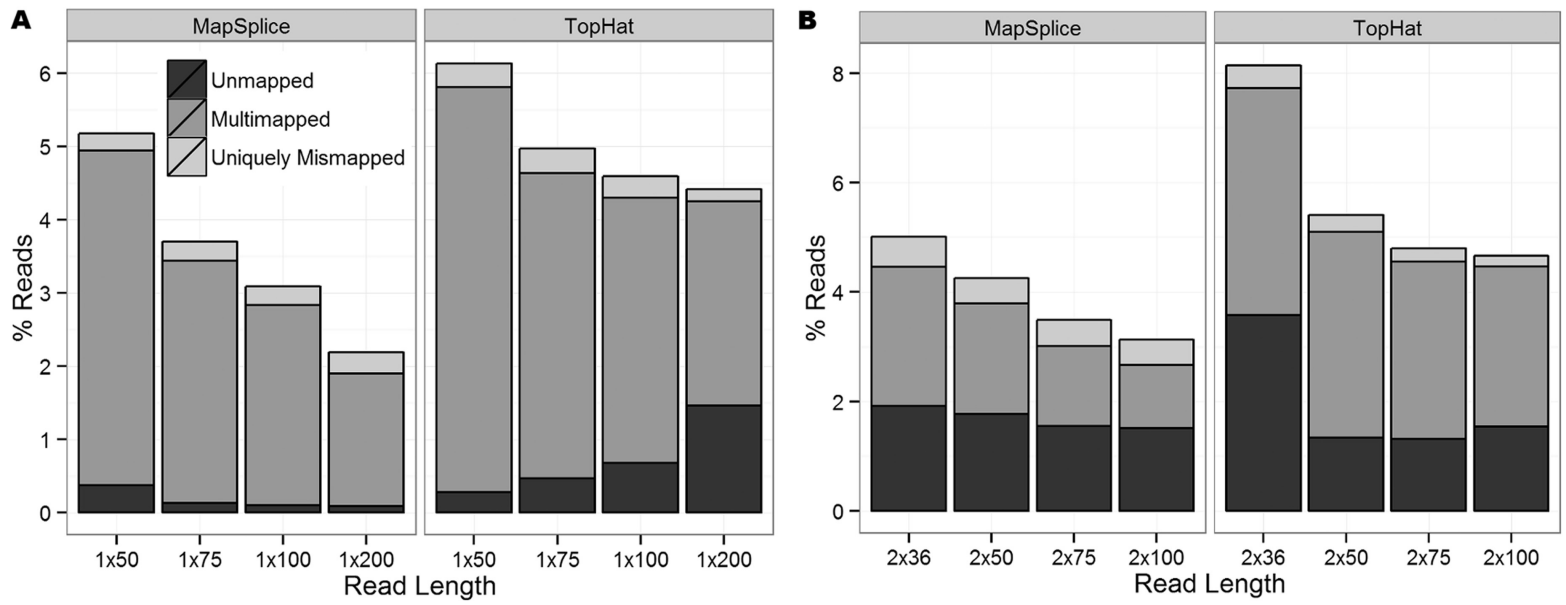
The proportion of unmapped, multimapped and uniquely mismapped reads for (**A**) all eight SE data sets and (**B**) all eight PE data sets. The remaining reads were correctly mapped.

**Figure 2.**
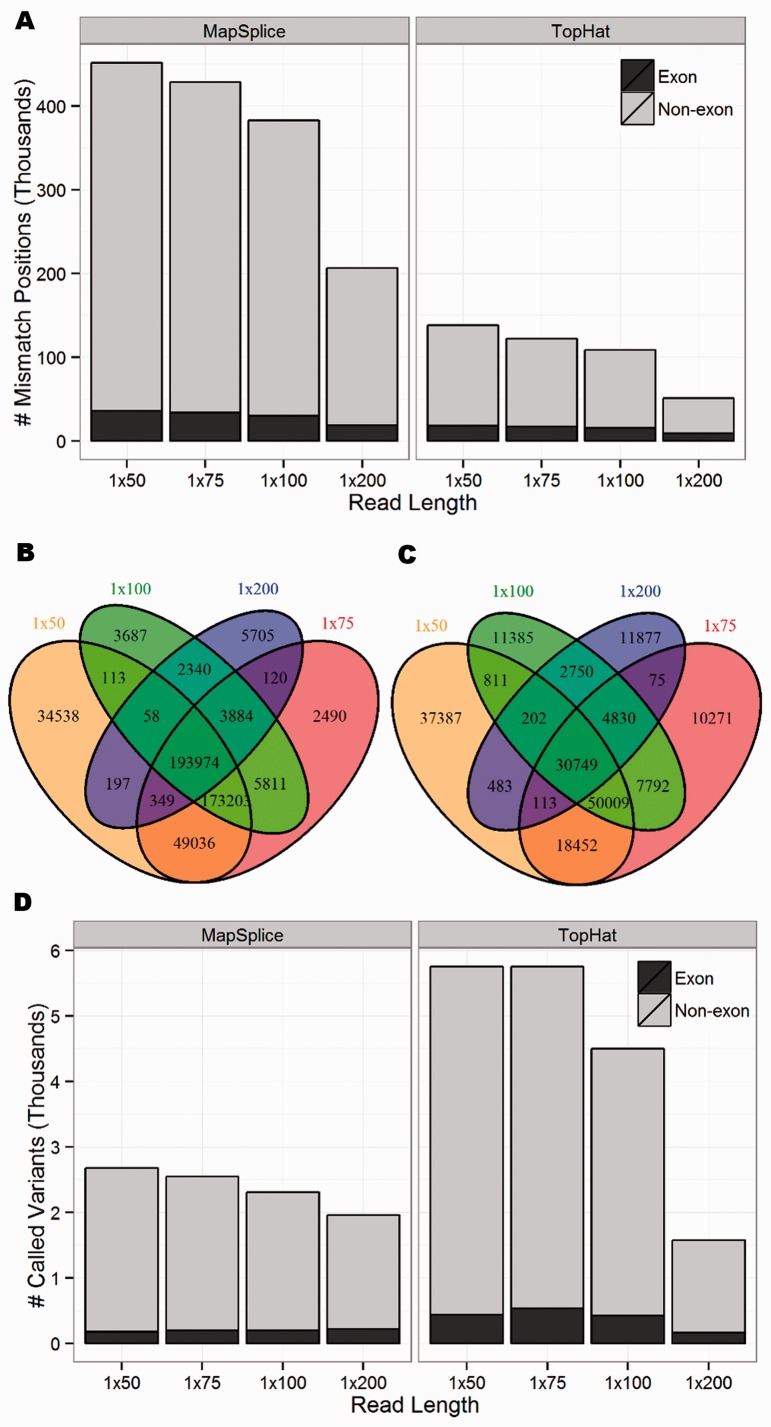
(**A**) The number of mismatch positions (SNDs) covered by at least one non-reference base for the eight SE data sets, where the number of exon positions is shaded black. The overlap of SNDs across the four SE data sets aligned with (**B**) MapSplice and (**C**) TopHat, showing that these positions are highly dependent on read length. (**D**) Total number of called variants, where the number of exon positions is shaded black. Although MapSplice has a larger number of SNDs, TopHat has more called variants.

For both SE and PE reads, the proportion of correctly mapped reads (reads that mapped uniquely to the correct genomic location) increased as read length increased (Figure 1). In general, TopHat reported more ‘unmapped reads’ (reads that failed to map to the genome) and ‘multimapped reads’ (reads that mapped to multiple locations in the genome) than MapSplice. Both aligners reported a similar number of ‘uniquely mismapped reads’ (reads that mapped uniquely to the wrong genomic location). To further understand these mapping errors and their influence on variant calling, attention was restricted to uniquely mapped reads because multimapped reads are often filtered out before calling variants as no more than one of these mapped locations can be correct (14,15). Following the terminology of (31), we define a ‘single nucleotide difference (SND)’ as a genomic position covered by at least one non-reference (mismatch) base. Figure 2A shows the number of SNDs for the SE data sets. Even though the MapSplice alignments reported more correctly mapped reads than TopHat (higher sensitivity), they also had approximately three times as many SNDs (lower specificity). While a large proportion of these positions occurred outside of known exons and can be easily filtered, tens of thousands of these SNDs still occurred in exons. Similar patterns were observed in the PE data sets (Supplementary Figure S1).

For each aligner, the SNDs were highly dependent on the read length (Figures 2B and C). For example, only 60.7% of all SNDs were identified across all four MapSplice SE data sets, while 14.5% were unique to a single read length. A similar pattern occurred when considering SNDs in exons only, with 42.1% common to all data sets and 12.5% unique to a single data set (Supplementary Figure S2).

Focusing on genomic positions with high coverage, we chose to call variants using VarScan, requiring 30× coverage with at least 10 reads supporting the non-reference allele. Because we did not insert errors or true SNPs into our reads, every read should perfectly map without any mismatches. Therefore, any variants that are called must be false positives due entirely to mapping errors. Each SE data set called over 1500 variants with ∼10% of these calls occurring in exons (Figure 2D). For three of the SE read lengths, approximately twice as many variants were called in TopHat than MapSplice. Because TopHat and MapSplice had roughly the same number of uniquely mismapped reads (Figure 1) and MapSplice had approximately three times as many SNDs, this suggests that the mismapped TopHat reads were piling up at fewer positions, resulting in higher coverage of the alternate base and, therefore, more called variants.

These mapping results demonstrate that, even in the absence of sequencing errors and SNPs, RNA-seq alignment algorithms have difficulty correctly mapping each read. We hypothesized that these mapping errors are likely due to sequence homology or difficulty aligning reads that span a splice junction. We found that, if correctly mapped, 100% of the uniquely mismapped SE reads would cover a splice junction (Supplementary Table S1). This proportion significantly dropped for multimapped reads, with <35% of MapSplice and <60% of TopHat multimapped reads originating from a region that spans a splice junction. This indicates that splice junctions increase alignment accuracy. Sequence homology demonstrated a smaller role in mapping errors. The proportion of uniquely mismapped reads mapping to a paralogous gene ranged from 3–5% for TopHat and 5–25% for MapSplice, with longer read lengths more likely to mismap to a paralog (Supplementary Figure S3).

To better understand the sources of mapping bias against spliced reads, we examined more closely a number of the uniquely mismapped SE reads. As evidenced by the *PABPC* gene family, it appears that both alignment algorithms assign a higher penalty to a read mapping to a splice junction with no mismatches than the same read mapping to a region without a splice junction but with a mismatch (Supplementary Figure S4). Many of the SNDs identified in our analysis were also observed in two glioblastoma cell line replicates (Supplementary Figure S4C), providing strong evidence that these mapping errors are present in real data sets and must be accounted for before drawing biological conclusions about novel variant positions.

We also aligned the four SE data sets to the set of UCSC transcripts (the transcriptome) using TopHat. Overall, the transcriptome alignments resulted in more accurate mappings and fewer SNDs than the genomic alignments (Supplementary Figure S5). These improved mapping results are expected because transcriptome alignments do not have the difficult task of identifying splice junctions. Although transcriptome alignments generally do a better job of correctly mapping reads that span known splice junctions, the results presented here may be overstating the accuracy of transcriptome alignments because we aligned to the same transcriptome from which reads were simulated.

In a typical sequencing experiment, thousands of bases will be incorrectly called due to sequencing error. To incorporate this type of technical error, we randomly inserted sequencing errors at a rate of 10^−^^4^ to the 1 × 75 data set, resulting in >1.5 million reads (0.75% of all reads) containing errors. Reads containing random sequencing errors were more likely to be unmapped (0.49 versus 0.47%; one-sided binomial *P* = 0.006), more likely to be multimapped (4.24 versus 4.17%; *P* < 10^−^^4^) and less likely to be uniquely mismapped (0.23 versus 0.33%; *P* < 10^−^^15^). The uniquely mismapped reads were less likely to originate from a region that spans a splice junction (90.5 versus 100%; *P* < 10^−^^15^). Over 1.4 million additional SND positions were reported owing to the insertion of sequencing errors, which is over 11 times greater than the number of SNDs due to mapping errors. However, because the sequencing errors were randomly distributed throughout the transcriptome, these additional SNDs lacked the coverage to be called as variants. While some of these results may be surprising, such as a smaller proportion of reads being uniquely mismapped, these results are from a single application of simulating sequencing errors. Therefore, repeating this analysis may produce different results.

### Mapping-caused SNDs in variant databases

**Figure 3.**
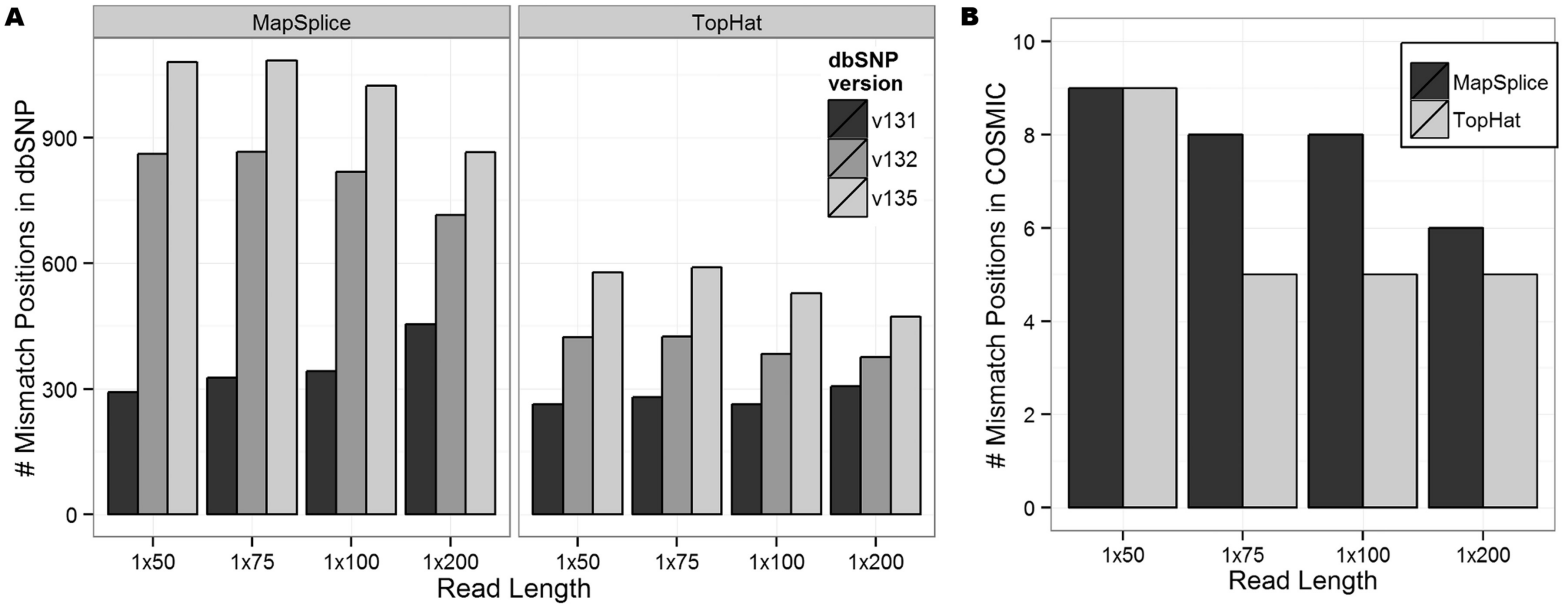
(**A**) The number of exon SNDs reported in dbSNP for the eight SE data sets. Each shaded bar represents a different version of dbSNP (131, 132 and 135). (**B**) The number of SNDs reported in COSMIC.

Several hundred reported exon positions in dbSNP overlapped the identified SNDs (Figure 3A). This represents <0.2% of all dbSNP positions in exons and 1–5% of all SNDs. Across all data sets, we observed a sharp increase in the number of SNDs across newer versions of dbSNP, coinciding with the rise in popularity of next-generation sequencing. A similar trend was observed in the PE data sets and the set of high-coverage variant positions called by VarScan (Supplementary Figures S6 and S7). Our simulations are based on RNA-seq, while the variants listed in dbSNP should be based on DNA sequencing projects. Therefore, we cannot say for certain that these published SNPs are false positives due to mapping errors. However, these positions should still be treated with caution because some of these mapping errors may be common to both RNA-seq and DNA sequencing reads.

Considering mapping-caused SNDs in the COSMIC database, >25 times fewer SNDs were present in COSMIC than dbSNP (Figure 3B), but this was expected, as COSMIC is smaller and more heavily curated. The small intersection between COSMIC and the SNDs suggests that the most promising mutation candidates are not likely to occur at SND positions. Additionally, many of the COSMIC positions identified in this analysis as probable mapping errors were present across multiple read lengths, with 67% (6/9) of these positions common to all SE MapSplice data sets and only 11% (1/9) unique to a single SE MapSplice data set (Supplementary Figure S8).

### Insertion of known SNPs

**Figure 4.**
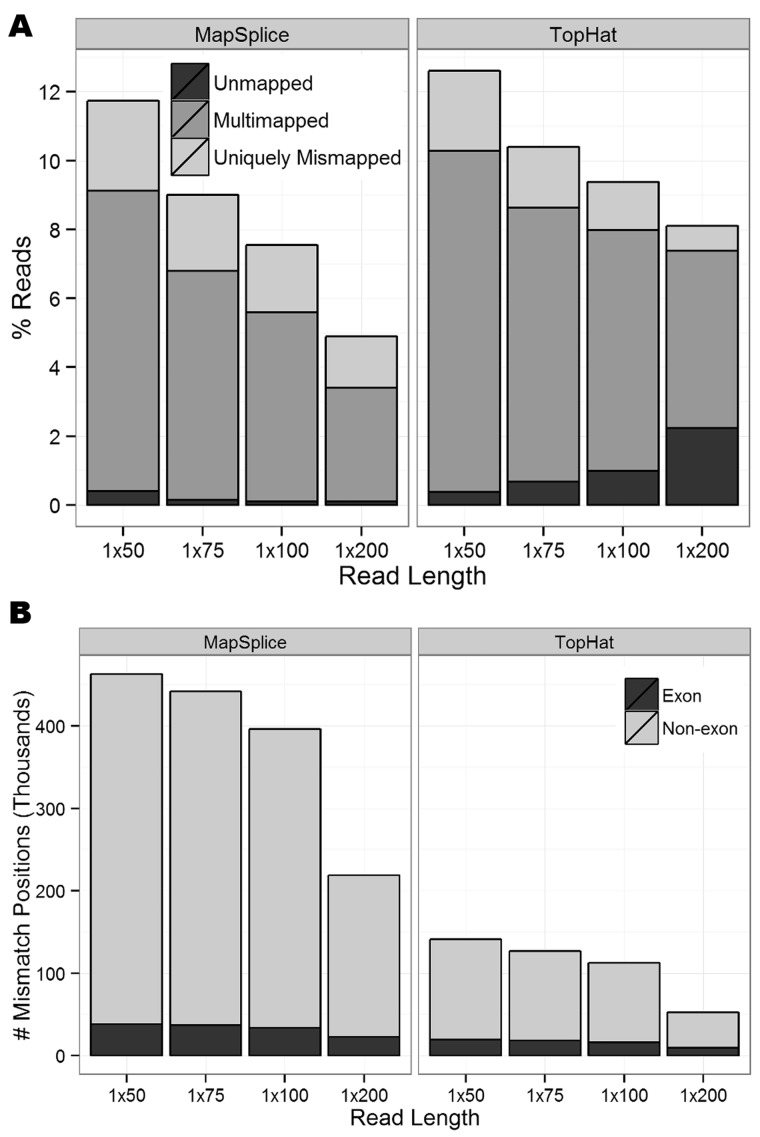
(**A**) The proportion of unmapped, multimapped and uniquely mismapped reads for all 8 SE data sets after manually inserting known SNPs from dbSNP. (**B**) The number of SNDs covered by at least one non-reference base, where the number of exon positions is shaded black.

So far, we have only investigated reads that perfectly match the reference sequence and reads with random sequencing errors, but we have not considered reads containing known SNPs. To modify our simulation to reflect this biological variability, we added 563 438 SNPs reported in dbSNP version 131 into the SE reads by editing the reference allele base to the SNP alternate allele base, and this substantially increased the number of mapping errors (Figure 4A). When comparing the alignments of SNP-inserted reads to the reference reads across all eight SE data sets, there was no significant difference in the percentage of unmapped reads (paired *t*-test *P* = 0.09), but significantly more SNP-inserted reads were multimapped (*P* < 10^−^^4^) and uniquely mismapped (*P* < 10^−^^4^). Of the uniquely mismapped reads, only 17–75% span a splice junction when correctly mapped, significantly less than the 100% observed in the reference reads (*P* < 10^−^^15^ for binomial test that the proportion equals one for each of the eight SE data sets; Supplementary Table S2). Conversely, significantly more uniquely mismapped reads mapped to a paralogous gene (23–33%, *P* < 10^−^^15^ for binomial test that the proportion of SNP-inserted reads mapping to a paralog is greater than the proportion of reference-only reads mapping to a paralog for each of the eight SE data sets). This demonstrates the difficulty that current alignment algorithms have with correctly mapping reads that contain a SNP in addition to those that span a splice junction.

When restricting attention to uniquely mismapped reads that mapped to exons, we identified several hundred additional SNDs from the SNP-inserted reads that did not occur at an inserted SNP position and were not previously discovered when considering reference-only reads (Figure 4B). As expected, a majority of these novel SNDs were aligner-specific (Supplementary Figure S9).

The previous analyses demonstrated that many SNP-inserted reads are mapping to an incorrect genomic location. Therefore, several of the inserted SNP positions that should have been identified in this analysis were missed. Depending on the read length, 4–11% of all inserted SNP positions failed to be covered by even a single read containing the alternate allele (Supplementary Figure S10A). These loci appear to be homozygous with the reference allele, when in fact they have a SNP and are heterozygous. These loci represent false negatives that cannot be overcome with additional sequencing. These results are consistent with the reference-allele bias previously described in RNA-seq (14).

### DNA-WES analysis

Next, we compared the RNA-seq results with DNA-WES. Because DNA-WES involves capturing genomic DNA on an exon array before high-throughput sequencing, the sequenced reads may contain some surrounding intronic sequence but, unlike RNA-seq, will never span an exon junction with the intronic region spliced out. Earlier we showed that 100% of the uniquely mismapped reads from the SE data sets derived from reads that span an exon splice junction when correctly mapped. Therefore, we expected DNA-WES reads to have better mappability than RNA-seq reads.

**Figure 5.**
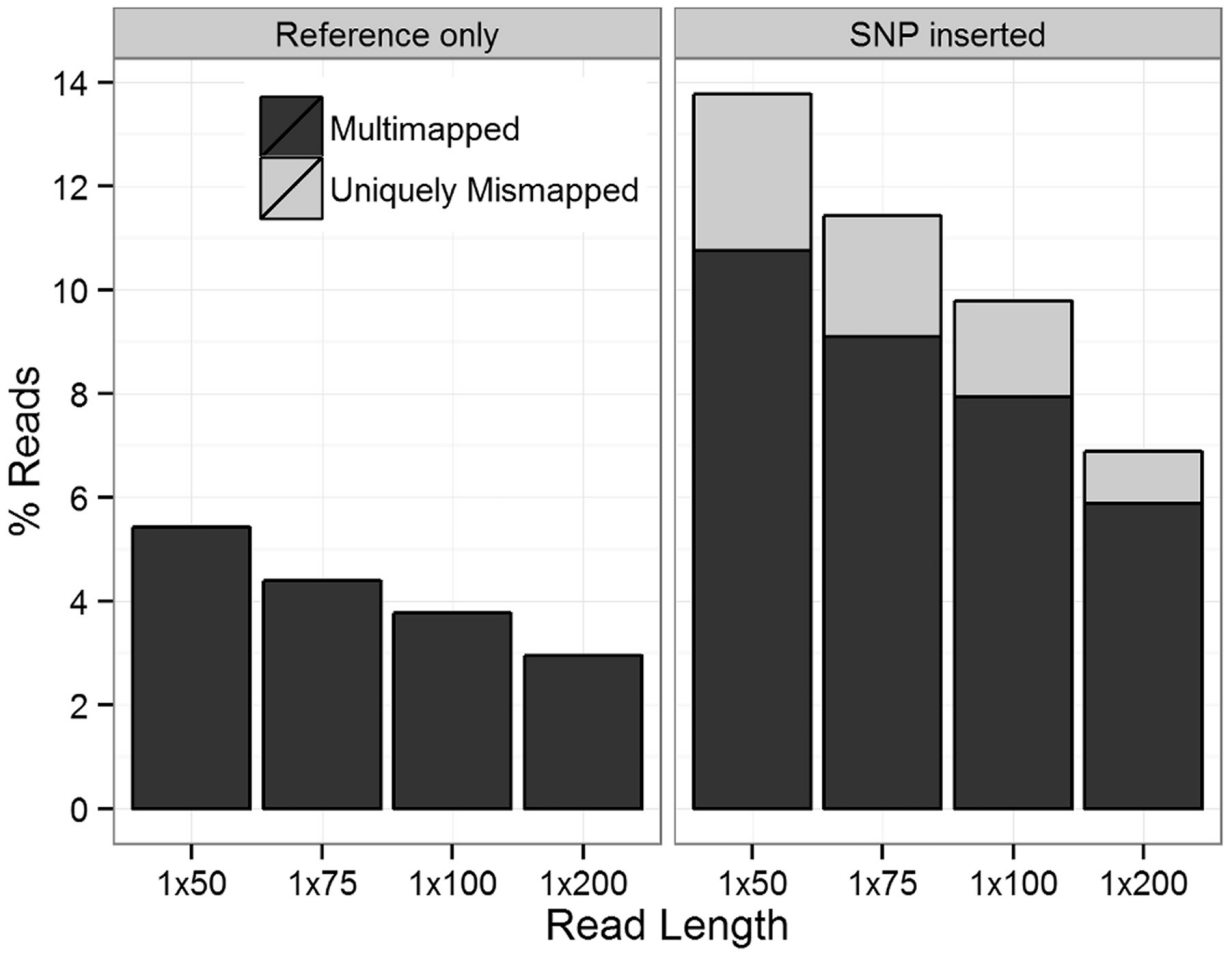
The proportion of multimapped and uniquely mismapped reads for the four DNA-WES data sets aligned with MapSplice that match the reference sequence (left) and have known SNPs from dbSNP manually inserted (right). None of the DNA-WES data sets have any unmapped reads.

Similar to the RNA-seq processing pipeline, we aligned the DNA-WES reads to the genome with MapSplice and TopHat. However, because no reads span splice junctions and both aligners use Bowtie (32) for alignment of unspliced reads, the results were nearly identical (slight inconsistencies are due to different filtering schemes and using different version of Bowtie). None of the DNA-WES reads were unmapped or uniquely mismapped (Figure 5A). The DNA-WES reads were slightly more likely to be multimapped than the RNA-seq reads (paired *t*-test *P* = 0.008). Additionally, there was not a single mismatch position (SND) for the DNA-WES reads across all SE read lengths.

The insertion of known SNPs into the DNA-WES reads resulted in 1–3% of reads being uniquely mismapped, on average equivalent to the proportion of uniquely mismapped RNA-seq reads (Figure 5B, *P* = 0.66). Surprisingly, this had only a small effect on the number of DNA-WES SNDs, as the number of SNDs was <50 for all eight data sets. This demonstrates the high specificity of DNA-WES and that sequence homology is solely responsible for the uniquely mismapped reads. DNA-WES recovered a significantly smaller proportion of the inserted SNPs than RNA-seq in six of the eight data sets (Supplementary Figure S10B; *P* < 8 × 10^−^^5^ for binomial test that the proportion of SNP-inserted reads recovered in DNA-WES is less than the proportion of SNP-inserted reads recovered in RNA-seq for six of the SE data sets. Insignificant results for both 1 × 50 data sets). These results suggest that RNA-seq may be more sensitive than DNA-WES, but we do not believe that is the case. Although not accounted for in our simulated data sets, in practice, RNA-seq experiments will yield less uniform coverage across the set of transcripts than DNA-WES, resulting in decreased sensitivity of RNA-seq compared with the results presented here.

### Blacklist filtering

Our next goal was to see how many of the SNDs are present in real RNA-seq data. We combined the SNDs identified by BlackOPs into a ‘blacklist’ to filter variant calls against. RNA-seq data for two replicates of the U87 glioblastoma cell line were aligned with MapSplice. A total of 2924 and 2341 variants were called by VarScan in exons, with 2129 variants identified in both replicates. A common filtering step when identifying novel variants is to remove previously reported population-level SNPs (16,27). As we have shown, this filtering will potentially filter out some false positives due to mapping errors because these databases are likely to contain false variants. Of the 2129 variants, 2000 were listed in dbSNP version 135, leaving 129 unannotated variants (Supplementary Table S3). To further reduce the number of potential false positives due to mapping errors, we filtered out an additional 92 SNDs (91 from the 1 × 75 MapSplice SE blacklist and one from the 1 × 75 SNP-inserted blacklist) for a final set of 37 variants. Accounting for mapping errors through this simple filtering scheme removed 71.3% (92/129) of the novel candidates as indistinguishable from mapping artifacts. This set of 129 potential novel candidates was significantly enriched for SNDs (binomial *P* < 10^−^^15^).

There exist additional analysis tools, such as the BAQ option in SAMtools (28), which aim to improve variant calling by correcting for mapping errors. For comparison, we implemented the BAQ adjustment and removed variant positions listed in dbSNP, and this left 102 variants. There were 30 variant positions that passed both the BAQ adjustment and blacklist filtering schemes (Supplementary Figure S11). This suggests that filtering SNDs listed in our blacklist is a more stringent filter than BAQ.

A previous study (27) performed WGS followed by variant calling on the same U87 cell line. Of the 129 unannotated RNA-seq variants, 7 (5.4%) were also called as variants in the WGS data, 6 of which remained after blacklist filtering. The one WGS variant that was filtered by BlackOPs was also filtered by BAQ and mapped to the region shown in Supplementary Figure S4C. This variant is listed in dbSNP versions 132 and 137 (rs201081849) but was removed from dbSNP version 135 and has not yet been validated, suggesting that this WGS variant may also be an artifact of mapping error. Owing to low coverage of WGS, many of the remaining RNA-seq variants are likely to be genomic variants missed in WGS. For example, 6 of the 129 RNA-seq variants had moderate supporting evidence (at least 2 WGS reads matching the alternate allele) but were not called as variants in WGS owing to low coverage (covered by <15 reads). All six of these variants remain in the blacklist-filtered set. After removing the six genomic variants and six variants with supporting WGS evidence, 25 unannotated RNA-seq variants remained in the blacklist filtered set. The majority (86%) of these remaining variants had low coverage in the WGS data (seven fail to be covered by even a single read and an additional 17 covered by <15 reads). Therefore, true genomic variants may exist at these positions, but the lack of WGS coverage makes it difficult to determine. The lone remaining variant, which is covered by 26 WGS reads, is a potential candidate for RNA editing. However, as this transcriptional event is rare and this variant is not of the common A-to-I (G) editing (3), it is more likely a false positive.

## DISCUSSION

Researchers have been aware of mapping errors since the early days of sequencing. Labs that regularly process large amounts of high-throughput sequencing data are likely to have investigated such errors and developed processing steps to mitigate their impact on analyses. However, there exists a lack of comprehensive work describing RNA-seq mapping errors and their downstream effects. We have presented evidence that even in the ‘best-case scenario’—no SNPs, no sequencing errors and high coverage—RNA-seq mapping errors are widespread. We observed that millions of reads spanning splice junctions will be uniquely mapped to an incorrect genomic location with a mismatch. As expected, reads containing known SNPs have a much higher rate (up to 6×) of being uniquely mismapped. Although not tested here, one can image the further decrease in mappability when incorporating sequencing errors and indels.

BlackOPs, a publicly available tool that uses simulated transcript reads and outputs a blacklist of positions and alleles caused by mismapping, allowed us to fully characterize observed mapping errors. These results should motivate all labs interested in calling variants using high-throughput sequencing data to take additional precautions to reduce the number of potential false positives due to mapping errors. BlackOPs allows users to repeat the analysis presented here, modifying the parameters to match the read length, alignment algorithm and genome/transcriptome under study. While we have only focused on the human genome here, BlackOPs can be extended to genomes of other organisms. Variant lists can easily be filtered against the identified blacklist positions, as we performed on the cell line data. These precautions will help researchers to focus attention and resources on mutations most likely to be disease-related. Additionally, this could help develop curated versions of variant databases such as dbSNP, which is greatly needed as the false-positive rate has been estimated to be as high as 17% in dbSNP (33).

Mapping errors are responsible for large numbers of false positives when comparing person-to-person (germline) or tumor-to-normal (somatic). When looking across multiple samples, the same systematic mapping errors are likely to occur across all samples. Under sufficient coverage, these positions are more likely to be called as germline than somatic variants. However, cases exist where mapping errors can lead to false-positive somatic variant calls, making blacklist filtering necessary. For example, suppose that gene A has high expression in tumor and low expression in normal and gene B is expressed at relative levels in both tumor and normal. If reads from gene A (which is only expressed in tumor) are incorrectly mapping to gene B with a mismatch, it will falsely appear that a somatic variant is present in gene B in the tumor population.

We compared our method with the BAQ option in SAMtools and we noted that our blacklist method removes many more potential mapping errors than the BAQ adjustment, but further experimental validation is required to determine the false-positive and false-negative rates of the two methods. The Genome Analysis Toolkit (34) is another popular tool for mitigating the impact of mapping errors when calling variants, but it does not support RNA-seq experiments, so we were unable to compare with BlackOPs.

Between the two sequencing technologies that we evaluated, DNA-WES exhibited far fewer mapping errors and SNDs than RNA-seq. This difference was solely due to the presence of reads spanning splice junctions in RNA-seq because the simulation was otherwise identical. This points out the difficulty of gapped alignment in RNA-seq compared with ungapped alignment of DNA-WES. A relatively short read split by an intron becomes even shorter, and the number of locations that the read fragment could align to increases. Our results show that DNA-WES has the advantage of fewer SNDs compared with RNA-seq but other factors beyond our analysis of false positives should inform the decision on which sequencing technology to adopt in a study, such as target limitations. One drawback of DNA-WES is that it measures only a fixed set of known protein-coding exons. While a large majority of causal variants and driver mutations fall within these regions, DNA-WES will miss possibly important variants in other regions, such as a highly recurrent mutation in the promoter region of *TERT* in melanoma tumors (35,36).

There are some limitations to our study. Our study is not meant to be a comparison between RNA-seq alignment algorithms. For an in-depth comparison of RNA-seq aligners, see Grant *et al.* (2). For this reason, we chose to use only two alignment algorithms for this study: TopHat and MapSplice. This study should not be used as a comparison between the two aligners because parameters were not optimized for comparison purposes. Instead, we chose to align with the default parameters as this is representative of how most labs will use these alignment tools. Additionally, alignment algorithms are continually releasing new versions, improving both speed and accuracy, so the versions used in this article may soon be obsolete. Finally, the comparison between the transcriptome and genome alignments of TopHat should be treated with caution. One drawback of transcriptome alignments is that they are dependent on the supplied annotation and are unable to discover novel transcripts, isoforms and splice junctions. Because we aligned to the same transcriptome that the reads were generated from, the transcriptome alignment results were better than would be expected in practice.

We have shown that, among sixteen alignment models (algorithm plus read length), mapping errors will exist and that, in the absence of true SNPs, a majority of these errors will occur in reads that span splice junctions. These mapping errors are specific to the alignment algorithm and read length used. With the evidence provided here, we urge labs interested in RNA-seq variant calling to modify their pipelines and incorporate BlackOPs to account for possible mapping errors, as this simple step will greatly reduce the number of false positives. This will save both time and money when experimentally validating candidate disease-related mutations.

## SUPPLEMENTARY DATA

Supplementary Data are available at NAR Online, including [37].

## FUNDING

National Institutes of Health [U24 CA143848 to J.S.P., C.M.P. and D.N.H., U24 CA143848-02S1 to C.R.C. and D.N.H., F32 CA142039 to M.D.W.]. Funding for open access charge: NIH.

*Conflict of interest statement*. None declared.

## Supplementary Material

Supplementary Data
