# Supplementary material for: BlackOPs: increasing confidence in variant detection through mappability filtering
Source: Nucleic Acids Res. 2013 Aug 8;41(19):e178. doi: 10.1093/nar/gkt692 (PMC3799449; doi:10.1093/nar/gkt692)
Supplement: Supplementary Data [file supp_41_19_e178__index.html]

BlackOPs: increasing confidence in variant detection through mappability filtering — BlackOPs: increasing confidence in variant detection through mappability filtering — Supplementary Data 

# BlackOPs: increasing confidence in variant detection through mappability filtering

## Supplementary Data

files

**Files in this Data Supplement:**

- Supplementary Data - pdf file
