# Supplementary material for: BlackOPs: increasing confidence in variant detection through mappability filtering
Source: Nucleic Acids Res. 2013 Aug 8;41(19):e178. doi: 10.1093/nar/gkt692 (PMC3799449; doi:10.1093/nar/gkt692)
Supplement: Supplementary Data [file supp_gkt692_nar-01220-met-n-2013-File007.pdf]

SUPPLEMENTARY MATERIALS

This file contains Supplementary Tables 1-3, Supplementary Figures 1-11, and reference (37) to accompany the manuscript "BlackOPs: Increasing Confidence in Variant Detection through Mappability Filtering" by Cabanski *et al*.

Supplementary Tables 2

Supplementary Figures 4

References 13

SUPPLEMENTARY TABLES

		Uniquely Mismapped	Multimapped
**MapSplice**	**1x50**	494,960 (100)	9,303,120 (11.35)
	**1x75**	520,620 (100)	6,677,616 (18.37)
	**1x100**	527,116 (100)	5,453,942 (24.32)
	**1x200**	564,777 (100)	3,497,951 (33.58)
**TopHat**	**1x50**	655,567 (100)	11,250,948 (23.08)
	**1x75**	659,111 (100)	8,417,385 (33.54)
	**1x100**	586,609 (100)	7,234,411 (41.39)
	**1x200**	325,822 (100)	5,382,641 (58.14)

Supplementary Table 1. The number of uniquely mismapped and multimapped reads from the single-end datasets. The percentage of reads that should span a splice junction when correctly mapped is reported in parentheses.

		% Spanning Splice Junction	% Mapping to Paralog
**MapSplice**	**1x50**	26.32	24.35
	**1x75**	40.21	26.27
	**1x100**	51.08	28.64
	**1x200**	75.11	32.91
**TopHat**	**1x50**	17.56	23.34
	**1x75**	25.74	23.94
	**1x100**	32.15	24.56
	**1x200**	47.97	25.61

Supplementary Table 2. The percentage of uniquely mismapped reads from the SNP-inserted single-end datasets that spans a splice junction when correctly mapped or that maps to a paralog.

Filtering	Number Filtered	Remaining Variants
Exon variants called in both replicates		2129
Reported in dbSNP version 135	2000	129
Listed in MapSplice 1x75 blacklist	91	38
Listed in MapSplice 1x75 SNP-inserted blacklist	1	37

Supplementary Table 3. Summary of the BlackOPs filtering process to remove artifact variants that are indistinguishable from mapping errors. The additional filtering steps suggested here removed 71.3% of novel variants (92/129) as likely false positives due to mapping errors.

SUPPLEMENTARY FIGURES

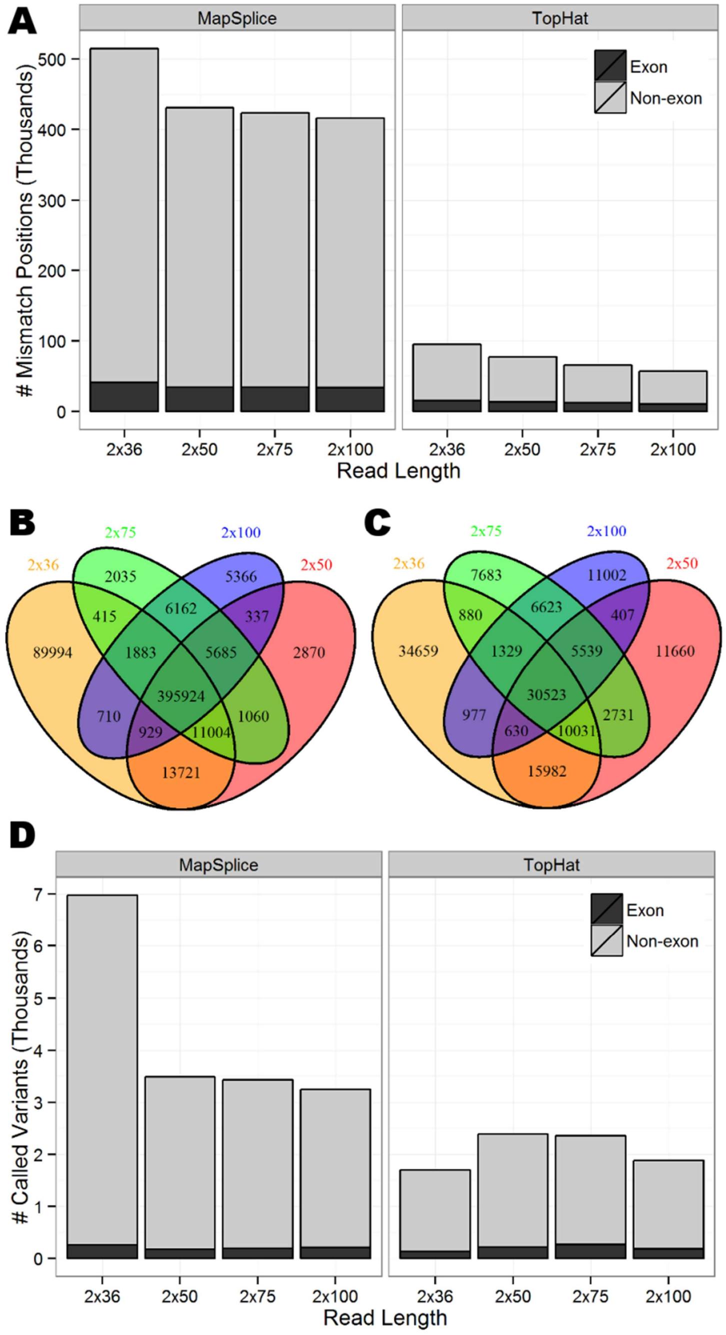

Supplementary Figure 1. (A) The number of mismatch positions (SNDs) covered by at least one non-reference base for the 8 paired-end datasets, where the number of exon positions is shaded black. The overlap of SNDs across the four paired-end datasets aligned with (B) MapSplice and (C) TopHat, showing that these positions are highly dependent on read length. (D) Total number of called variants, where the number of exon positions is shaded black.

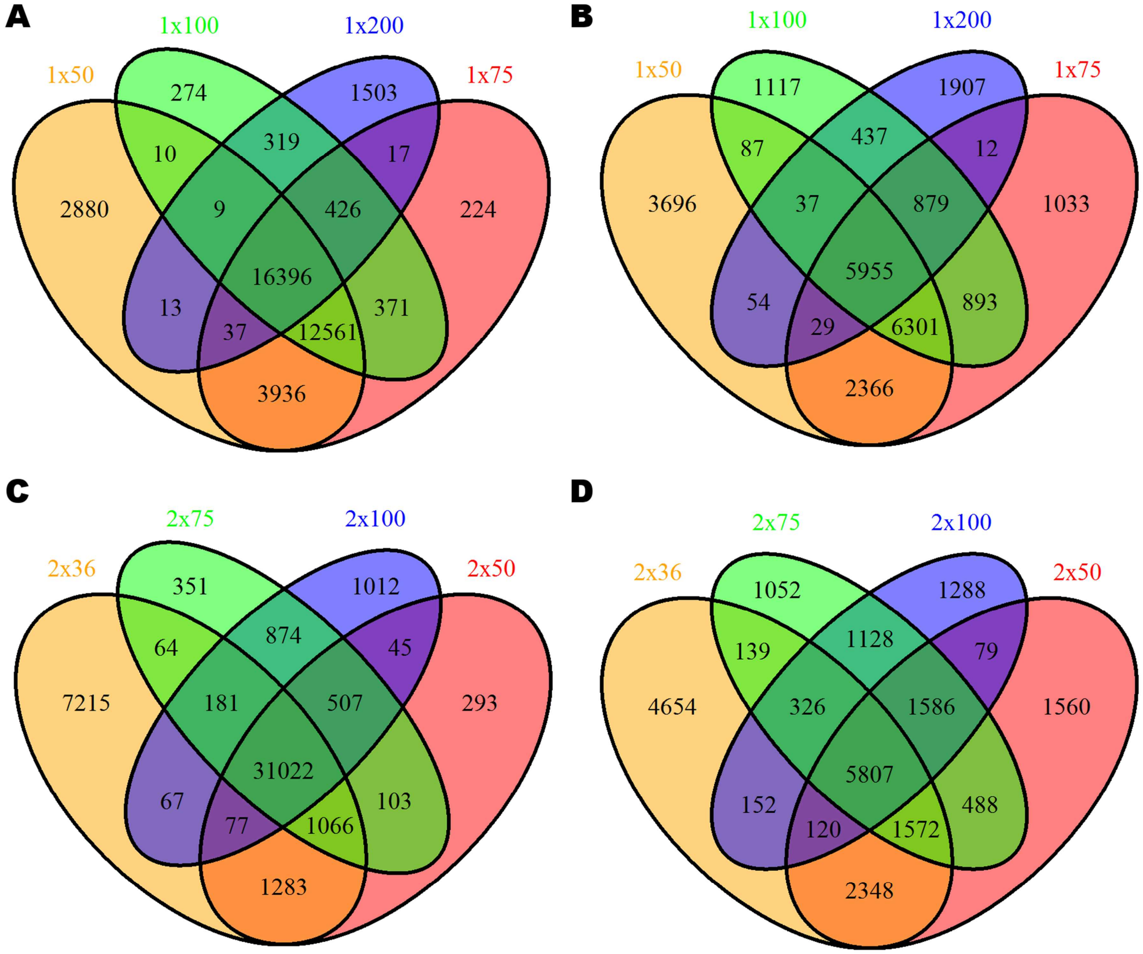

Supplementary Figure 2. The overlap of SNDs in exons across the four single-end datasets aligned with (A) MapSplice and (B) TopHat, and the four paired-end datasets aligned with (C) MapSplice and (D) TopHat. For each aligner, the SNDs are highly dependent on the read length.

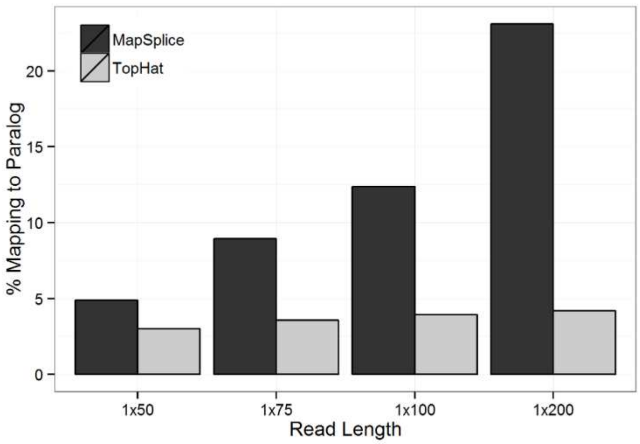

Supplementary Figure 3. The percentage of uniquely mismapped reads mapping to a known paralog for all 8 single-end datasets.

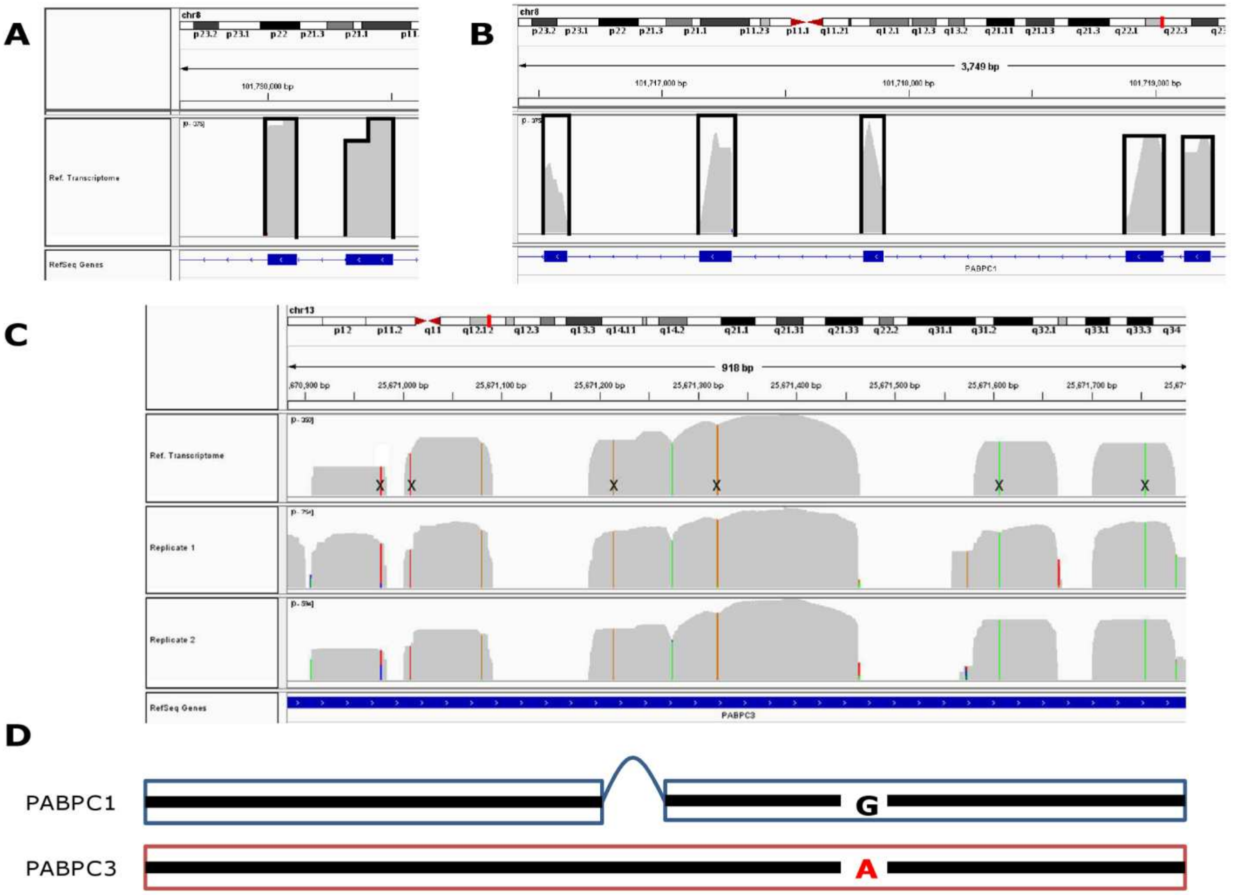

Supplementary Figure 4. Example of junction-spanning reads mapping uniquely to a paralogous gene with MapSplice (1x75 dataset). (A) Integrative Genomics Viewer (37) visualization of unique reads mapping to the correct location on PABPC1. Almost all reads originating from these exons map correctly, as the coverage (grey histograms) reaches the expected number of reads (black box). The step pattern observed in the second exon is due to multiple distinct isoforms. (B) Another region of PABPC1 where coverage is lower than expected, especially at the ends of exons. (C) Top row: uniquely mismapped reads mapping to paralog PABPC3 that should have mapped to PABPC1 (coverage reported on log scale). These reads should have spanned one of the splice junctions shown in panel B, but instead a mismatch is tolerated (designated by a colored line). Mismatch positions marked by an “x” (6 of 8) are reported as variants in dbSNP version 132. Bottom two rows: a similar pattern of coverage and non-reference bases is observed in two U87 cell line replicates. (D) Schematic of one region in PABPC1 (chr8:101,721,804-101,724,623) where originating reads should span a splice junction, denoted by a gap, but instead map to a region in PABPC3 (chr13:25,671,275-25,671,464) with a mismatch and no splice junction.

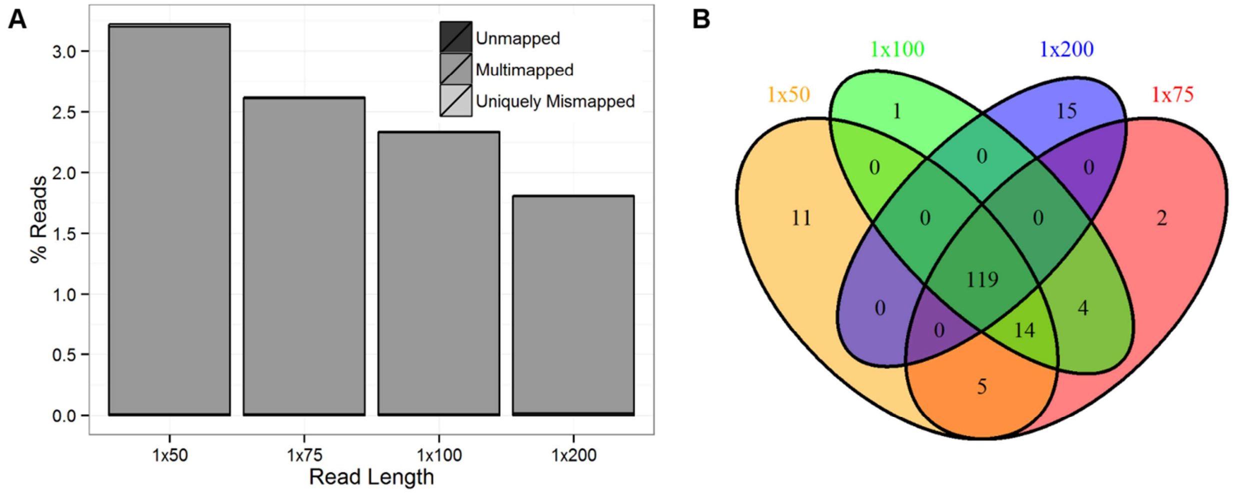

Supplementary Figure 5. (A) The proportion of unmapped, multimapped and uniquely mismapped reads for the 4 single-end datasets aligned to the transcriptome. (B) The overlap of SNDs across the four single-end datasets aligned to the transcriptome.

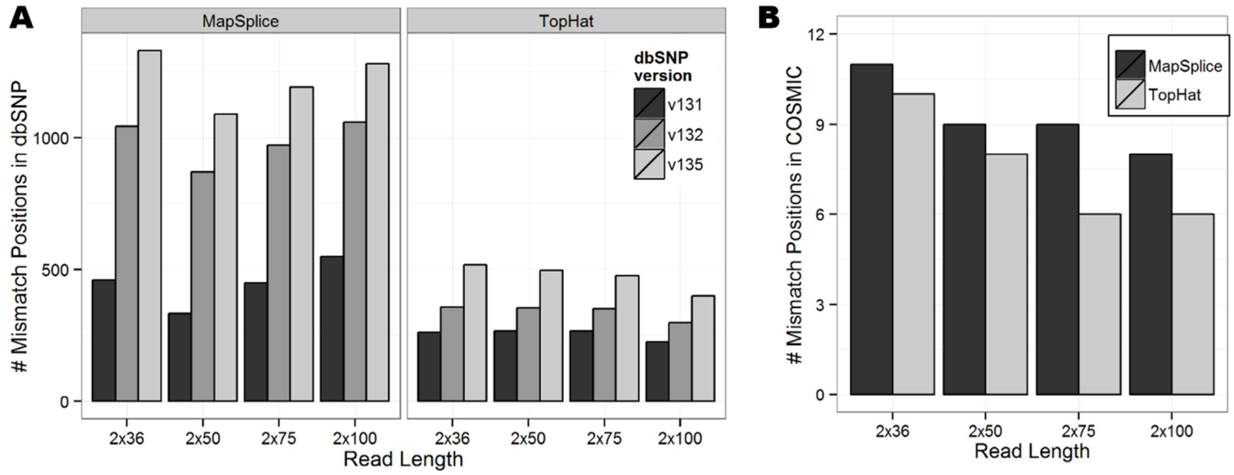

Supplementary Figure 6. (A) The number of exon SNDs reported in dbSNP for the 8 paired-end datasets. Each shaded bar represents a different version of dbSNP (131, 132 and 135). (B) The number of SNDs reported in COSMIC.

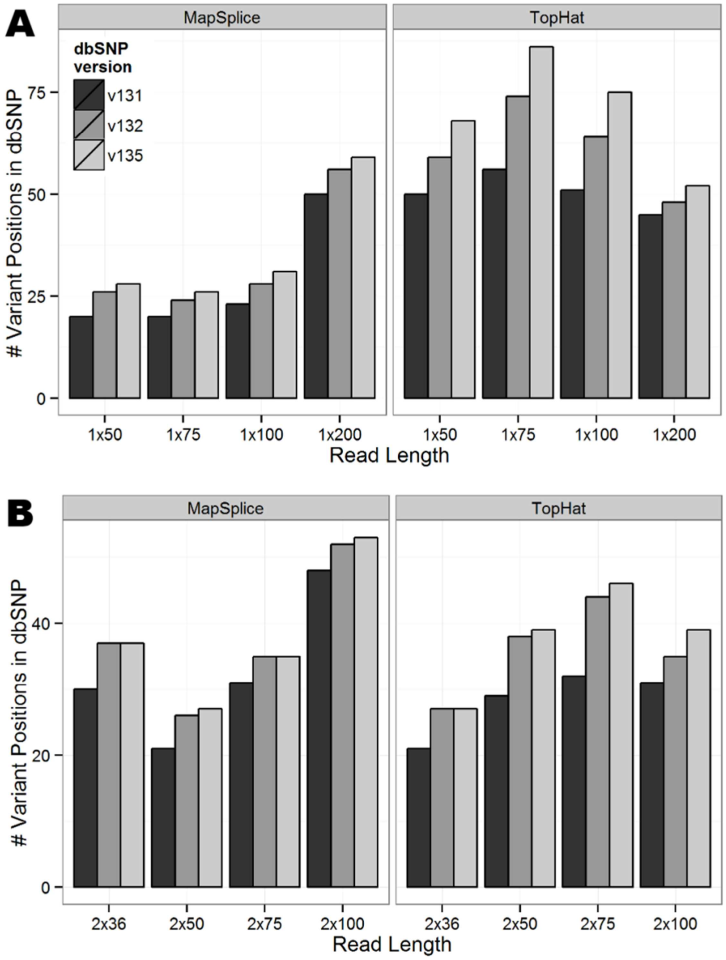

Supplementary Figure 7. (A) The number of called variant positions in exons reported in dbSNP for the (A) 8 single-end datasets and (B) 8 paired-end datasets. Each shaded bar represents a different version of dbSNP (131, 132 and 135).

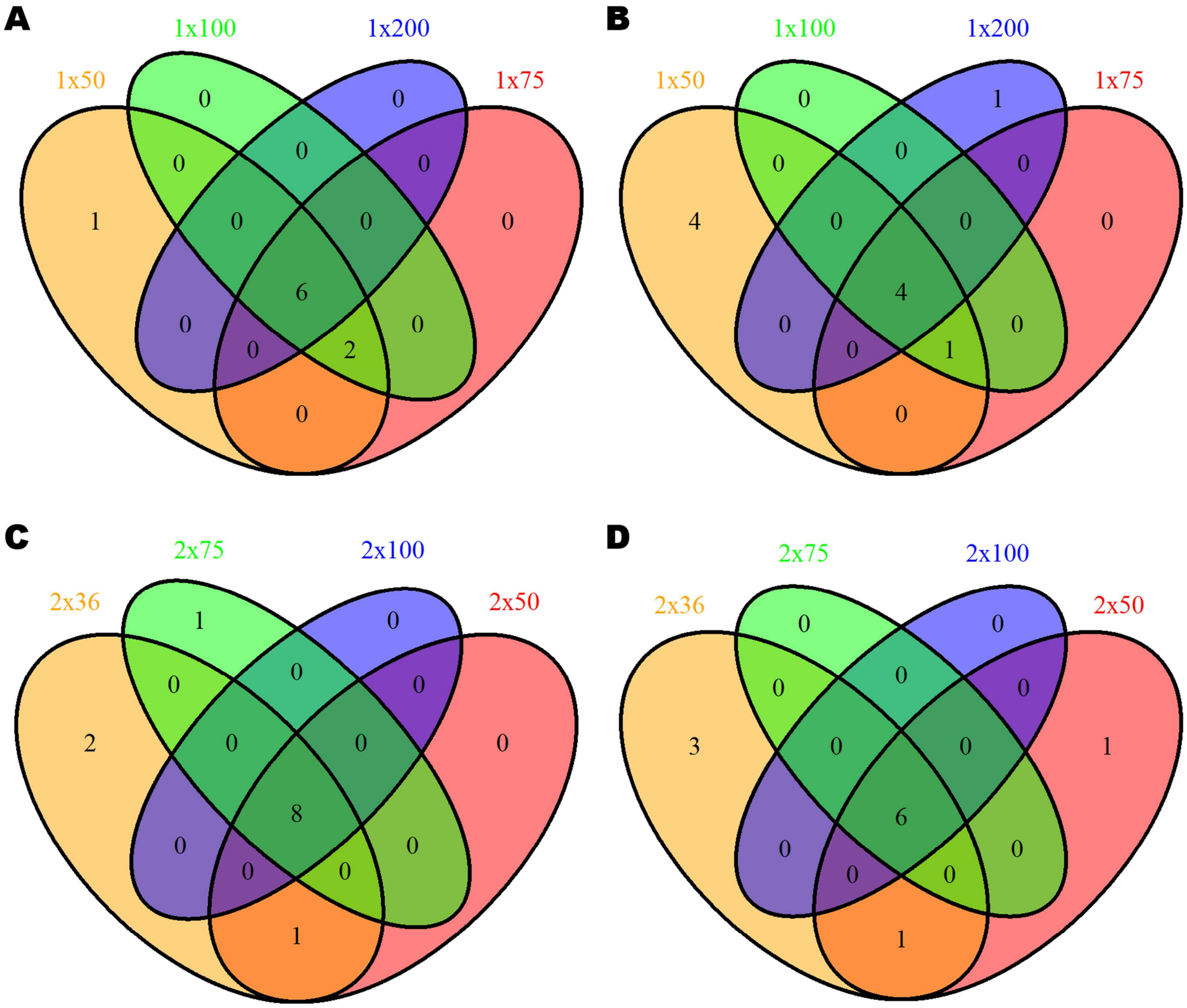

Supplementary Figure 8. The overlap of SNDs listed in COSMIC across the four single-end datasets aligned with (A) MapSplice and (B) TopHat, and the four paired-end datasets aligned with (C) MapSplice and (D) TopHat.

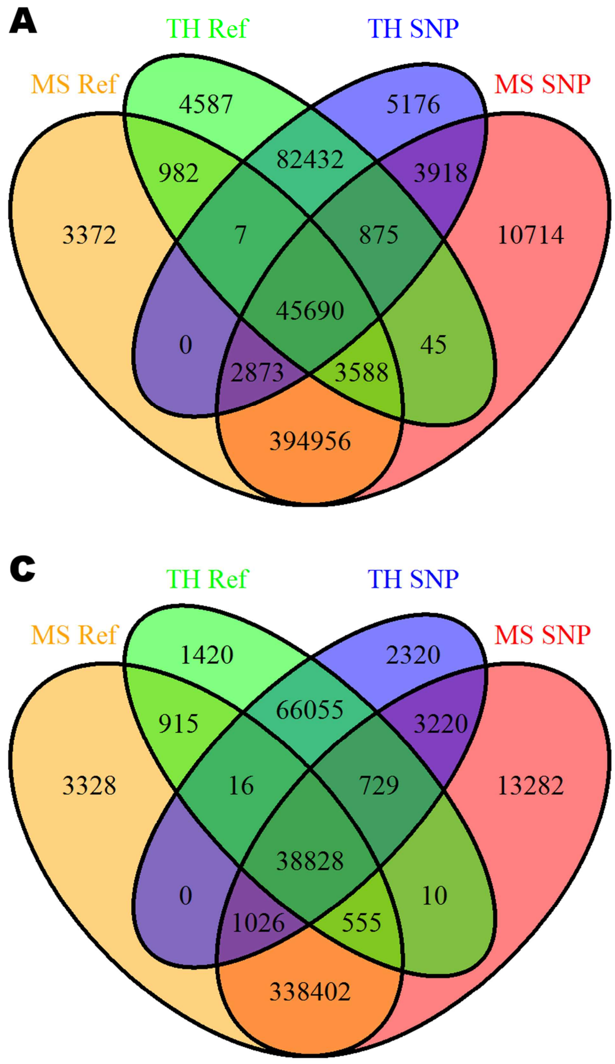

Supplementary Figure 9. The overlap of SNDs between reference-only reads aligned with MapSplice (MS Ref) and TopHat (TH Ref) and SNP-inserted reads aligned with MapSplice (MS SNP) and TopHat (TH SNP). (A) 1x50, (B) 1x75, (C) 1x100, (D) 1x200. Inserting known SNPs helps identify many novel SNDs, a majority of which are unique to the alignment algorithm used.

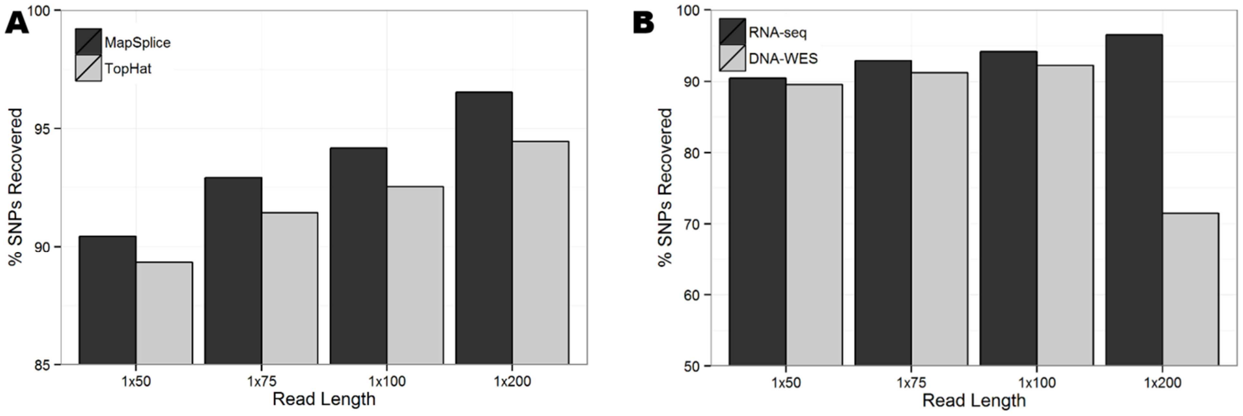

Supplementary Figure 10. The proportion of inserted SNPs recovered (covered by at least one read containing the alternate allele). (A) RNA-seq single-end reads. (B) RNA-seq and DNA-WES single-end reads aligned with MapSplice.

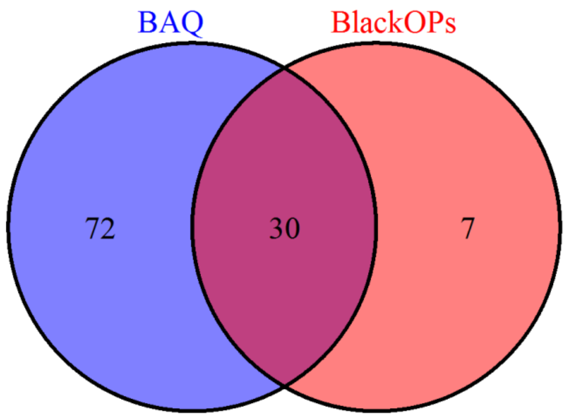

Supplementary Figure 11. Number of novel variants identified in the U87 cell line analysis that pass the BAQ and BlackOPs filters. BlackOPs filtered more potential variants as being indistinguishable from mapping artifacts than BAQ.

REFERENCES

37. Robinson,J.T., Thorvaldsdóttir,H., Winckler,W., Guttman,M., Lander,E.S., Getz,G. and Mesirov,J.P. (2011) Integrative genomics viewer. *Nat. Biotechnol.*, **29**, 24–26.
